# The promotion effect of carbon quantum dots derived from maize stalks on the photosynthesis of maize growth

**DOI:** 10.1186/s40643-026-01138-w

**Published:** 2026-09-30

**Authors:** Shuaijie Wang, Xiuping Li, Lihong Guo, Yanfang Li, Baohe Li, Mingcai Zhang, Jie Duan, Qi Dong, Libo Han, Caixia Di

**Affiliations:** 1 Inner Mongolia Academy of Agricultural & Animal Husbandry Sciences, Hohhot, 010031 China; 2 School of Physical Science and Technology, Inner Mongolia University, Hohhot, 010021 China

**Keywords:** Carbon quantum dots, Hydrothermal method, Chlorophyll content, Photosynthesis rate

## Abstract

This study described the preparation of water-soluble carbon quantum dots (CQDs) from maize stalks via a hydrothermal method at 200 °C for 10 h. The prepared CQDs exhibited an average particle size of 2.03 nm with a graphene structure, and the surfaces contained hydroxyl and carboxyl functional groups. For the aqueous solution of CQDs at a concentration of 100 mg/L, the optimal excitation wavelength was 410 nm, and the maximum emission wavelength was 495 nm. By utilizing the down-conversion luminescence properties of CQDs to enhance photosynthesis in maize growth, it was found that the optimal CQD concentration was 25 mg/L when maize seedlings were sprayed with CQDs at different concentrations for 7 days. The maize plant height, leaf area, leaf length, leaf circumference, chlorophyll content, Hill reaction activity, total photosynthetic rate, and net photosynthetic rate significantly increased by 43.2%, 25.6%, 31.2%, 27.3%, 35.2%, 45.5%, 29.8% and 46.6% compared with the CK, respectively. This work developed and demonstrated a feasible strategy for synthesizing cost-effective CQDs and may offer a potential benefit for early-stage corn seedling growth under controlled conditions.

**Graphical abstract:**

**Figure:**
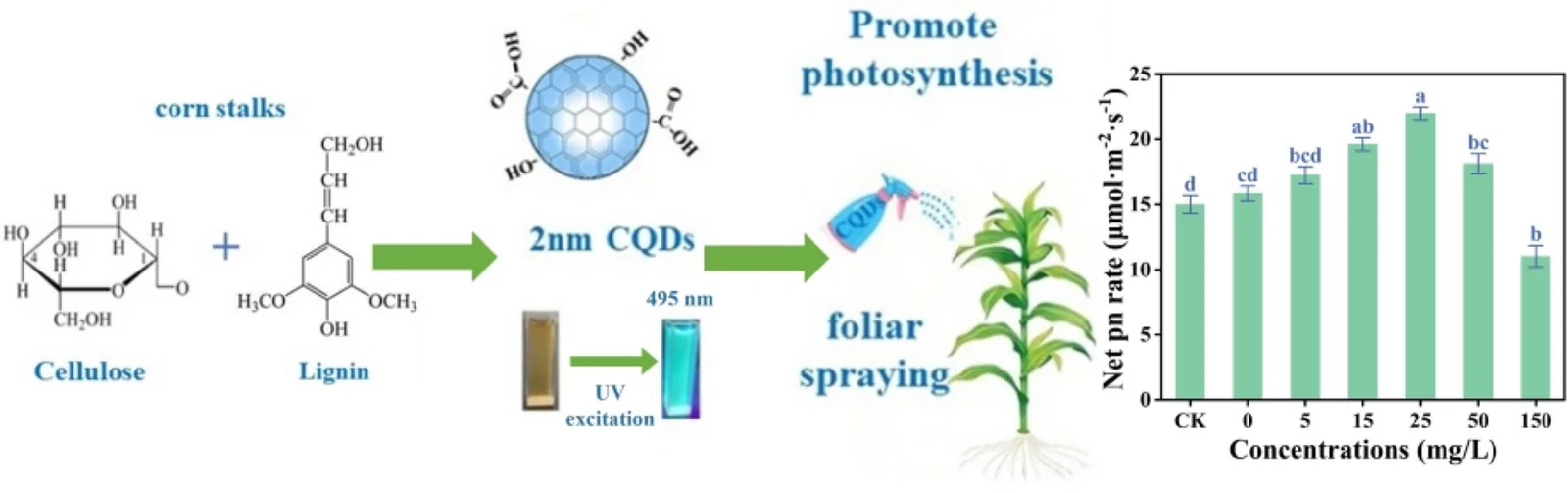

## Introduction

Carbon Quantum dots (CQDs) are zero-dimensional nanomaterials with dimensions smaller than 10 nm. Their core primarily consists of graphitized sp^2^ carbon atoms, exhibiting significant quantum confinement effects. This endows CQDs with unique physicochemical properties, including excellent optical performance, good biocompatibility, and non-toxicity (Chen et al. 2024; Jia et al. 2025; Liu et al. 2025; Sharma et al. 2025; Zhao et al. 2025). They demonstrate broad application prospects in fields such as fluorescence sensing (Viji et al. 2025), bioimaging (Ren et al. 2024), photocatalysis (Shi et al. 2025), drug delivery (Bhatia et al. 2024), mechanical lubrication (Yang et al. 2024a), and solar cells (Koç Keşir et al. 2025). CQDs with different microstructures, fluorescence properties, and surface functional groups have been successfully synthesized using chemical reagents as raw materials through various methods, including hydrothermal/solvothermal synthesis (Shabazi and Dall 2023), microwave-assisted synthesis (Tran et al. 2025), template-assisted synthesis (Mo et al. 2022), arc discharge synthesis (Chao-Mujica et al. 2021), and electrochemical synthesis (Jawad and Abdullah 2024).

In recent years, CQDs have garnered increasing attention in agriculture. The research demonstrated the effects of CQDs on the growth and photosynthesis of hydroponically cultivated green mustard plants, elucidating the electron-transfer mechanism within the CQDs/chloroplast complex (Chowmasundaram et al. 2025). Nitrogen-doped CQDs with down-conversion properties that convert ultraviolet light (380 nm) from the solar spectrum into blue light (450–475 nm) usable by microalgae, and found that the nitrogen-doped CQDs significantly enhanced Chlorella Sorokiniana growth and photosynthetic pigment accumulation (Liang et al. 2025). The other research demonstrated significant increases in photosynthetic rates across all stages when CQDs were applied via foliar spraying to laboratory-cultured peanuts (Dhiman et al. 2024). Although research on the application technology of CQDs has made considerable progress, some issues remain. One of the main problems is that the high cost of CQDs synthesized from these chemical raw materials hinders their large-scale application.

The preparation of CQDs from agricultural waste has received growing interest as a prominent research strategy (Astari et al. 2024; Meng et al. 2023; Wang 2024). Maize stalks are primarily composed of cellulose, hemicellulose, and lignin, which render them suitable as a raw material for synthesizing low-cost CQDs (Feng et al. 2025; Fu et al. 2023). Therefore, this route for CQDs synthesis offers low cost, industrial scalability, and high economic efficiency while effectively addressing pollution issues associated with agricultural wastes such as maize stalks (He et al. 2017; Zhao et al. 2020). From a technical perspective, the hydrothermal method provides lower costs, simpler procedures, and shorter synthesis times, making it more suitable for industrial-scale production (Mu et al. 2025; Xu et al. 2025). The studies have documented various applications of biomass-derived carbon quantum dots (CQDs) within the agricultural sector, including N-CQDs synthesized from whole wheat flour to enhance maize photosynthesis and to upregulate genes associated with carbon assimilation (Haider et al. 2024). Additionally, CQDs derived from coffee grounds have been shown to improve the net photosynthetic rate of rice by 32.66% (Thonghlueng et al. 2026). CQDs sourced from lawn waste have been utilized to promote the growth of Bermuda grass under saline-alkali conditions (Gan et al. 2026). Furthermore, various biomass carbon dots have been employed to augment crop photosynthesis through mechanisms such as light conversion and nutrient supply (Cheng et al. 2023).

In this study, CQDs were synthesized from corn stalks via a hydrothermal method. The formation mechanism and structure–property relationships of the CQDs were elucidated through analyses of their microstructure and optical properties, including morphology, particle size, surface functional groups, elemental composition, and fluorescence characteristics. Furthermore, to investigate the impact of CQDs on maize photosynthesis, foliar sprays of CQDs aqueous solutions at varying concentrations were applied. A systematic evaluation was then conducted by analyzing key plant indicators, including growth parameters, chlorophyll content, chloroplast activity, and photosynthetic rate. By correlating the properties of CQDs with the observed physiological effects, the potential mechanisms underlying the enhancement of photosynthesis were explored. This study seeks to elucidate the fundamental processes and physiological mechanisms by which carbon quantum dots (CQDs) influence photosynthesis in maize, thereby establishing a theoretical basis for the economical application of CQDs in crop improvement and nano-agricultural practices. Distinct from prior research, this investigation lies in the synthesis of CQDs from corn stalks and their pioneering application in modulating photosynthesis within the same crop, maize.

## Experimental methods

### Synthesis and characterization of CQDs

CQDs were synthesized from maize stalks via a one-step hydrothermal process (Mu et al. 2024; Zhao et al. 2026). Dried maize stalks were ground and sieved through a 60-mesh screen. A 0.3 g sample was mixed with 20 mL of ultrapure water and 10 mL of anhydrous ethanol. The well-mixed suspension was transferred into an autoclave and subjected to hydrothermal treatment at 200 °C for 10 h, yielding a CQDs precursor solution. The precursor solution was sequentially processed by filter paper filtration, microfiltration (0.22 μm), dialysis (1000 Dalton for 6 h), and drying (at 50 ℃) to obtain CQDs powder ultimately. A typical batch synthesis starting from 0.3 g of maize stalk powder yielded approximately 5 mg of CQDs powder.

The morphology and size of CQDs were characterized using a transmission electron microscope (TEM) (FEI F20), with average particle size calculated via Image software. The crystal structure of CQDs was analyzed by X-ray diffraction (XRD) (D8 Advance). The surface functional groups of CQDs were investigated using a Fourier Transform Infrared spectrometer (FTIR) (Nicolet iS20). The elemental composition and chemical states of CQDs were determined by X-ray photoelectron spectroscopy (XPS) (Thermo Escalab 250Xi). Fluorescence spectra of the CQDs solution were tested using a photoluminescence spectrometer (PL) (FL 8500). The absorption spectrum of the CQDs solution was measured by a UV–Vis spectrophotometer (UV-3600). The electron transfer rate (ETR) was directly calculated using the fluorescence module of the Li-6800 photosynthesis system. Specifically, according to the instrument manual, the absorption ratio of light energy by maize leaves was set to 0.84, the energy distribution ratio for Photosystem II (PSII) was set to 0.5, and the photosynthetically active radiation (PAR) was set to 1000 μmol m^−2^ s^−1^. During testing, steady-state fluorescence (Fs) and maximum fluorescence under light-adapted conditions (Fm′) were recorded. Using these, the photochemical efficiency (ΦPSII) was calculated, and then the ETR value was derived using the formula ETR = ΦPSII × PAR × 0.84 × 0.5.

1 $$\Phi PSII = \frac{{\left( {Fm^{\prime } - Fs} \right)}}{{Fm^{\prime } }}$$

2 $$ETR = \Phi PSII \times PAR \times 0.84 \times 0.5$$

3 $$\frac{Fv}{{Fm}} = \frac{{\left( {Fm - F0} \right)}}{Fm}$$

4 $$NPQ = \frac{{\left( {Fm - Fm^{\prime } } \right)}}{{Fm^{\prime } }}$$

5 $$Rate = \frac{{u\left( {C0 - Cs} \right)}}{A}$$

The data are shown as averages. We performed statistical analyses using a one-way analysis of variance (ANOVA), followed by a Tukey post-hoc test to compare multiple groups. We considered results significant when *P* was less than 0.05. All calculations were conducted using GraphPad Prism software. Biological replicates (n ≥ 3) are specified in the corresponding figure legends. All statistical analyses and plotting were performed using Origin software. The significance letters (a, b, c, …) on the figures are based on these analyses, and the corresponding *P*-values are indicated in the figure legends.

Quinine sulfate (dissolved in 0.1 M H_2_SO_4_) was used as the reference standard. During measurement, the absorbance of both the sample and the reference at the excitation wavelength was controlled below 0.05 to eliminate inner filter effects, and the quantum yield (QY) was calculated using the standard reference method.

The chromaticity coordinates and purity were both obtained using Origin 2023 software, utilizing the official OriginLab plugin Chromaticity Diagram.opx. The standard observer mode was set to CIE 1931 2°

To exclude the potential interference from residual inorganic nutrients derived from the biomass feedstock, the concentrations of K, P, and N in the final dialyzed CQDs solution (25 and 150 mg/L) were quantified. For the 25 mg/L CQDs solution, the K, P, and N contents were 0.220 mg/L, 0.010 mg/kg, and not detected, respectively; for the 150 mg/L CQDs solution, these values were 1.436 mg/L, 0.215 mg/kg, and not detected, respectively. These concentrations are considerably lower than the threshold levels typically required for observable physiological effects in plants, suggesting that the residual inorganic nutrients alone are unlikely to account for the photosynthetic enhancements observed in CQD-treated maize leaves. Accordingly, the positive effects reported in this study can be primarily attributed to the intrinsic properties of the CQDs rather than to nutrient artifacts.

### Effects of CQDs on maize plant growth

Maize seeds were purchased from Lihe Agriculture Company (variety Lihe No. 1). Greenhouse experiments were conducted in Hohhot, Inner Mongolia Autonomous Region, China. The cultivation period spanned July to August, featuring relatively moderate day and night temperatures and good light conditions. Maize seeds were evenly distributed in pots measuring 50 cm × 30 cm × 30 cm, with eight plants per pot. Four pots formed one group, totaling 7 groups. After 7 days of growth, foliar spraying with the CQDs solution was initiated. Maize leaves were sprayed daily with 5 ml of CQDs solution per plant for 12 days. Experimental groups received solutions at concentrations of 0, 5, 15, 25, 50, and 150 mg/L, while the control group (CK) received no spray.

Maize leaf photosynthetic rates and fluorescence analysis were measured using a photosynthesis analyzer (Li-6800). Maize leaf chlorophyll content was determined with a portable chlorophyll meter (CCM-300). Maize leaf size (area, length, perimeter) and health status were assessed by a root scanner (AM350). We employed the HEPENGBIO chloroplast extraction kit (non-enzymatic method) to extract intact chloroplasts suspended in buffer solution. Under light exposure, these chloroplasts release oxygen to reduce the 2,6-dichlorophenol (DCIP) dye. Upon dye reduction, the color changes from blue to colorless, allowing determination of chloroplast Hill reaction activity based on changes in solution absorbance (Cao & Zhou 2026; Scafaro et al. 2023). The relative electron transport rate of maize leaves was assessed using the HEPENGBIO chloroplast electron transport activity detection kit. The absorbance spectrum of the chloroplast solution was measured using a UV–vis spectrophotometer. Hydroxyl radical content was detected using the hydroxyl radical assay kit (Kopylchuk et al. 2023).

## Results and discussion

**Fig. 1:**
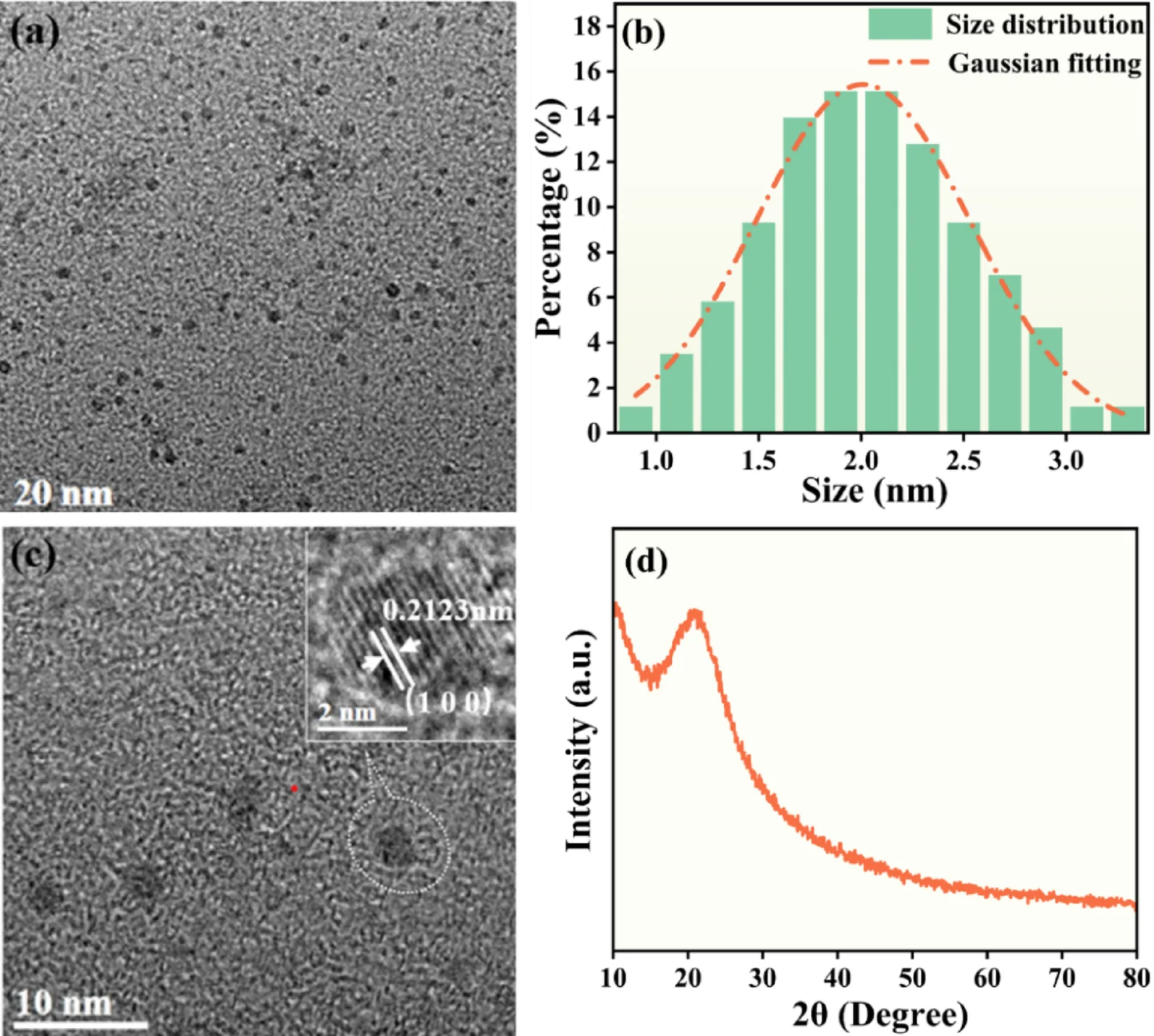
The microstructure of synthesized CQDs: **a** TEM image, **b** particle size distribution (100 randomly selected CQD particles), **c** lattice spacing, **d** XRD pattern

The morphology and average particle size of the CQDs samples were characterized using TEM, as shown in Fig. 1a. It can be observed that the CQDs synthesized from maize stalks exhibit spherical morphology with uniform particle size distribution and excellent dispersion. The particle size distribution in Fig. 1b indicated that CQDs follow a normal distribution ranging from 0.9 to 3.28 nm, with an average size of 2.03 nm. The HR-TEM inset in Fig. 1c shows ordered lattice striations on the CQDs surface with an interplanar spacing of 0.2123 nm, corresponding to the characteristic graphite phase (100) plane. This confirmed that the synthesized CQDs possessed a graphitic structure and local lattice ordering observed by HR-TEM. The XRD pattern in Fig. 1d revealed a diffraction peak at 22°, corresponding to the interface diffraction of the amorphous carbon structure of the CQDs. These results were in agreement with previous studies (Pal et al. 2020; Zhu & Hu 2021).

**Fig. 2:**
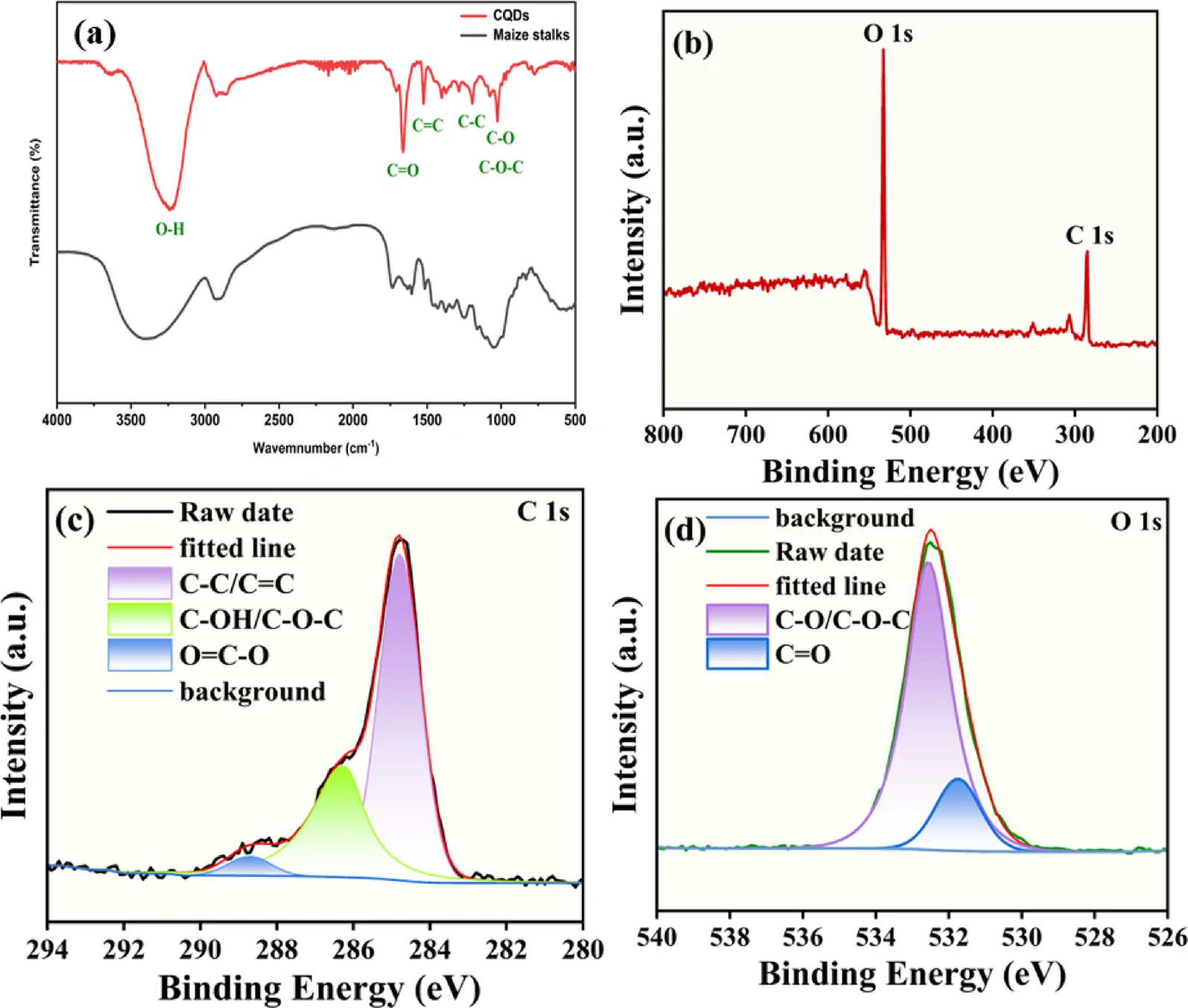
Surface state and elemental states of synthesized CQDs: **a** FTIR spectrum (CQDs and maize stalks), **b** XPS total spectrum, **c** C 1 s high-resolution spectrum, **d** O 1 s high-resolution spectrum

To quantitatively evaluate the structural transformation from raw maize stalks to CQDs, the FTIR spectra of both samples are shown in Fig. 2a. The disappearance of the C–O–C stretching vibration at ~1050 cm⁻^1^ and the attenuation of C–H stretching at 2920 cm⁻^1^ indicate cellulose and hemicellulose decomposition during hydrothermal carbonization. The enhanced C=O (1710 cm⁻^1^) and C=C (1620 cm⁻^1^) peaks confirm conjugated aromatic structures with surface carboxyl/carbonyl groups. After reaction, the weakening of absorption at 2920 cm⁻^1^ and 1730 cm⁻^1^ indicates degradation of aliphatic and ester bonds; the decreased and changed shape of the 1050 cm⁻^1^ peak suggests cleavage of glycosidic bonds and formation of small molecular frameworks. The O–H absorption at 3400 cm⁻^1^ remains, showing hydrophilic oxygen groups on the surface, consistent with Zeta potential results. The C=C peak at 1630 cm⁻^1^ indicates the graphitized carbon core. The XPS survey spectrum in Fig. 2b revealed characteristic peaks of C 1 s and O 1 s for the synthesized CQDs, located at 285.37 eV and 532.86 eV, respectively. In the fine spectrum (Fig. 2c), the C1s binding energy peaks at 284.80, 286.23, and 288.71 eV corresponded to C–C/C=C (peak area =3864), C–OH/C–O–C (peak area =2164), and O=C–O (peak area =235), respectively. Figure 2d indicates two predominant peaks in the O 1 s spectrum at 531.75 eV and 532.56 eV, corresponding to C=O (peak area =3004) and C−O/C–O–C (peak area =13,543) bonds, respectively. These results confirmed the presence of multiple functional groups, such as -OH, –COOH, and C=C on the CQDs surface. Meanwhile, these functional groups conferred CQDs with excellent dispersibility in water.

**Fig. 3:**
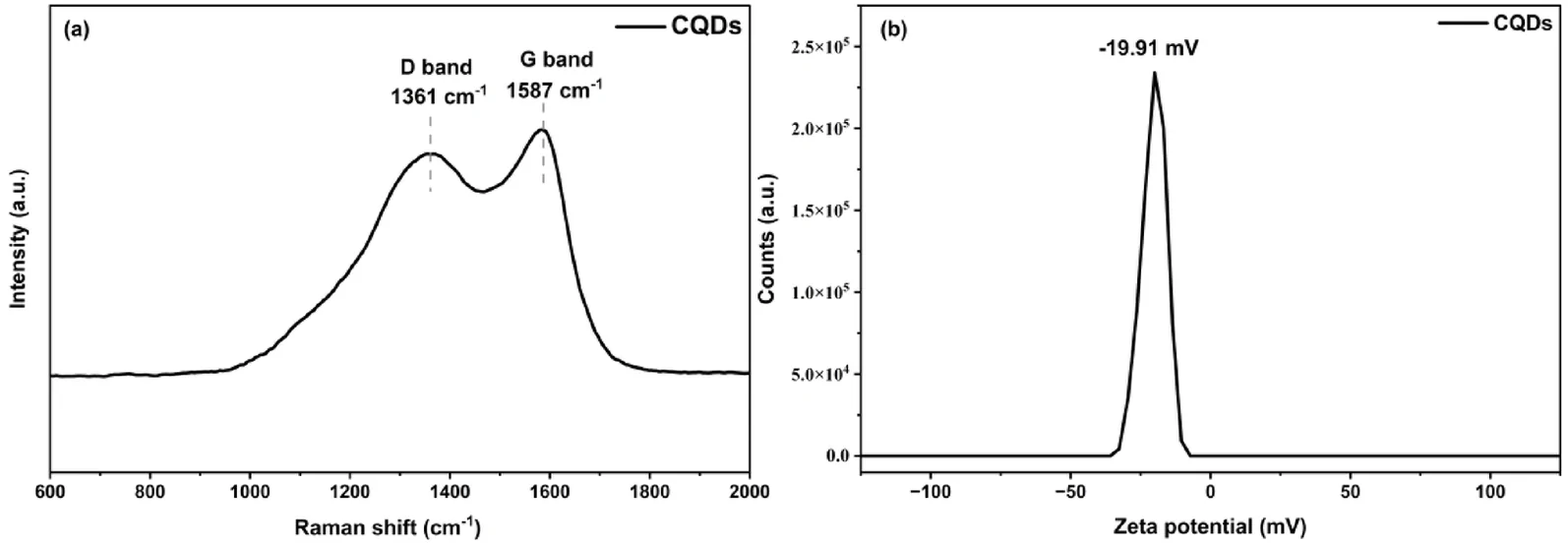
CQDs: **a** Raman spectrum and **b** Zeta potential spectrum

The Raman spectrum of the CQDs shows two bands at around 1361 cm^−1^ (D band) and 1587 cm^−1^ (G band). The D band indicates structural disorder, while the G band confirms graphitization, aligning with biomass-derived carbon dots. Figure 3b shows that the CQDs have a zeta potential of about − 19.91 mV, mainly from oxygen groups like –COOH and –OH, matching FTIR results (Fig. 2a). These groups aid dispersion in water and offer moderate stability. The single-peaked distribution suggests uniform charge and no impurities, which is important for biological and agricultural water applications.

**Fig. 4:**
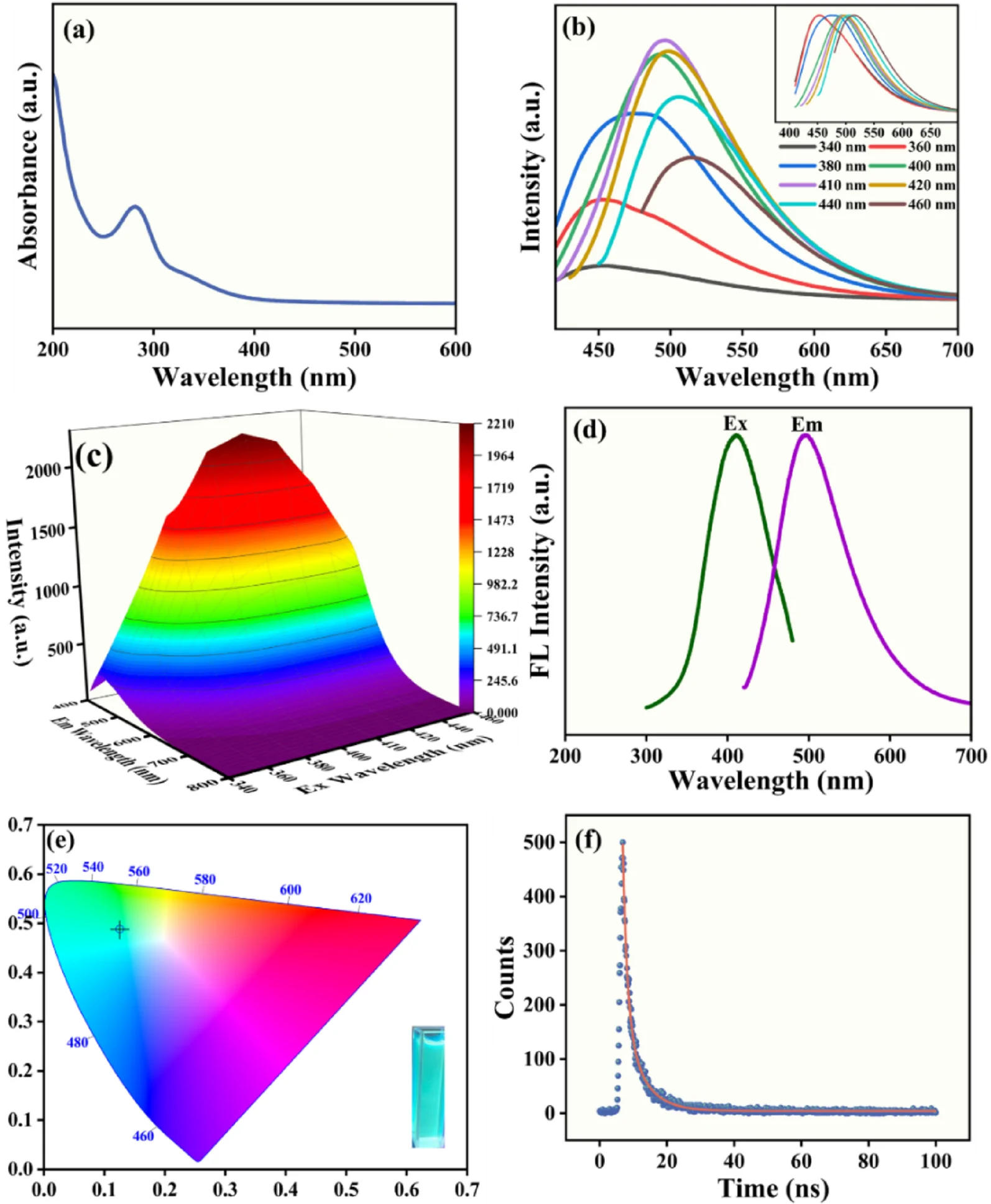
Optical Properties of synthesized CQDs: **a** Absorption spectrum, **b** Emission spectra under different excitation wavelengths, **c** excitation-emission matrix, **d** Optimal excitation wavelength and maximum emission wavelength, **e** CIE diagram of emission spectra, **f** Average fluorescence lifetime

Figure 4 shows the optical properties of the synthesized CQDs. As shown in Fig. 4a, the CQDs exhibited an obvious absorption peak in the ultraviolet region, with the maximum absorption peak wavelength at 285 nm. Figure 4b and c displayed the emission spectra of the prepared CQDs under excitation from 340 to 460 nm. The results show that the maximum emission intensity occurs at 495 nm when the excitation wavelength is 410 nm. As the excitation wavelength gradually increased from 340 to 460 nm, the emission wavelength of CQDs also redshifted. Figure 4d displayed the optimal excitation and emission spectra of CQDs. Figure 4e shows the CIE chromaticity diagram of the synthesized CQDs emission spectrum. The corresponding chromaticity coordinates and color purity are (0.13, 0.49) and 32.2%, respectively. The dominant wavelength of the CQDs was 505 nm, emitting cyan-blue light, which was consistent with the color observed by the naked eye. Figure 4f shows the fitted curve of the fluorescence lifetime of the CQDs. The average fluorescence lifetime was calculated to be 5.13 ns. The fluorescence quantum yield (QY) of the as-prepared CQDs was determined to be 2.86% using quinine sulfate as a reference. This value falls within the typical range for undoped biomass-derived CQDs reported in the literature. For instance, Blue flag flower-derived CQDs and cellulose/lignin-based CQDs have been reported with QYs of 7.41% and ~8.7%, respectively (Mohammed et al. 2025), whereas olive leaf-derived CQDs can reach up to 71.4% under specific high-temperature or doping conditions(Yang et al. 2024b). In the present study, the measured QY of 2.86% is comparable to that of pristine biomass CQDs without external heteroatom doping or surface passivation (typically in the range of 2.5–4.0%), confirming that the synthesized CQDs exhibit optical properties consistent with their undoped biomass-derived nature. The fluorescence lifetime and quantum yield of the synthesized CQDs were in line with those reported for most biomass-derived CQDs (Yang et al. 2024b). These results indicated that the as-prepared CQDs can be defined as a down-conversion luminescent material, which converts part of the ultraviolet light into cyan-blue light. The Optical properties may have a certain promoting effect on the photosynthesis of maize.

**Fig. 5:**
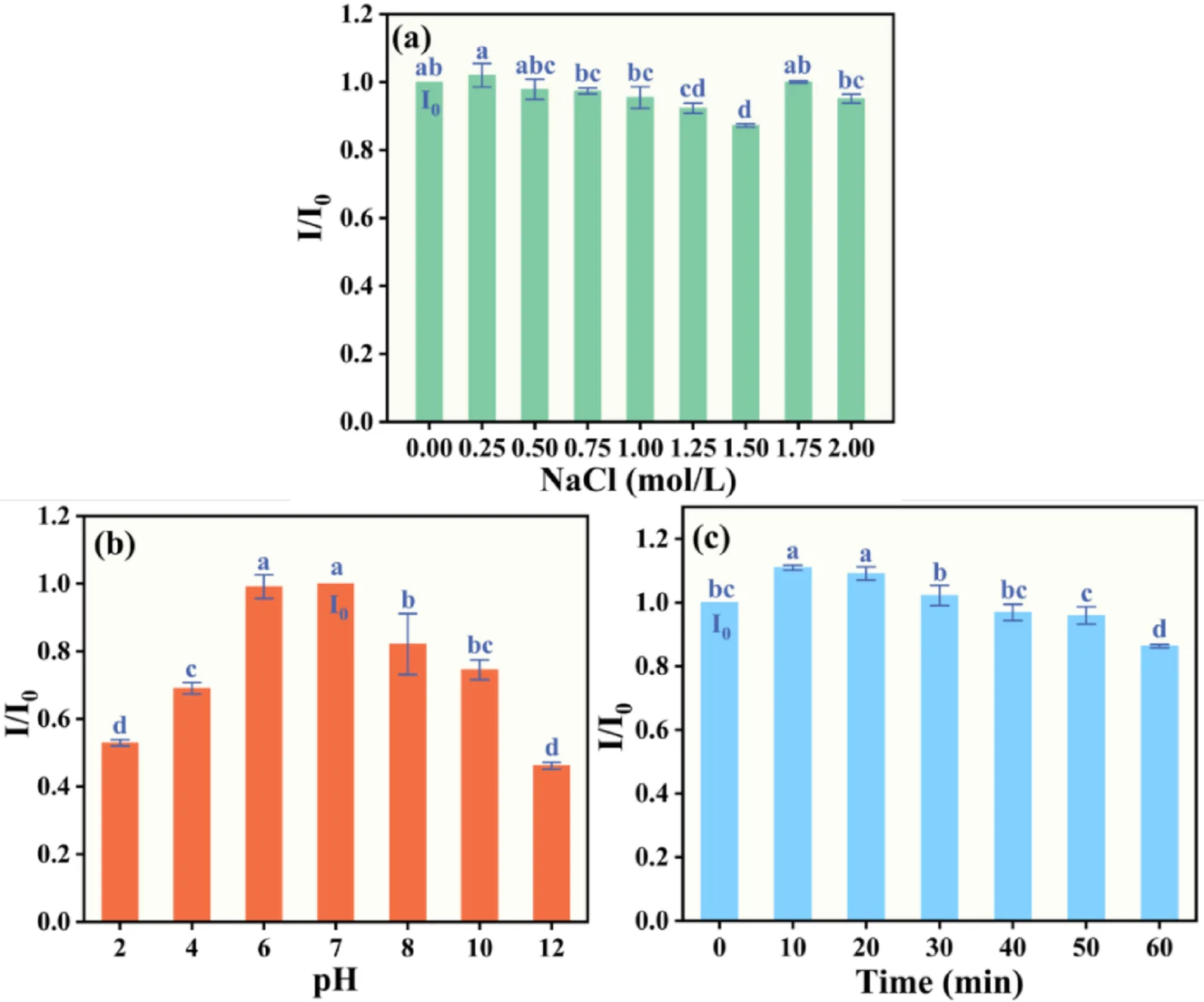
Fluorescence stability analysis of synthesized CQDs: **a** salt concentrations, **b** pH values, **c** UV light exposure times. Error bars correspond to SD (n = 3). Marked with different letters indicating a significant difference (*p* < 0.05)

The practical deployment of CQDs required sufficient optical stability under different environmental conditions. Figure 5a shows that as the NaCl concentration increased from 0 to 2 mol/L, the fluorescence intensity of CQDs remained essentially unchanged at higher ionic concentrations, indicating that ionic strength had no significant effect on the fluorescence of CQDs. Figure 5b shows that the fluorescence intensity of CQDs remained essentially unchanged in neutral solutions but significantly diminished in strong acidic or basic solutions. CQDs contain abundant functional groups, and it was speculated that their surface structure may have been disrupted in acidic or basic environments, leading to reduced fluorescence intensity. As shown in Fig. 5c, the fluorescence intensity of the CQDs remained stable even after 60 min of continuous irradiation at 365 nm UV light, indicating excellent resistance to photobleaching.

**Fig. 6:**
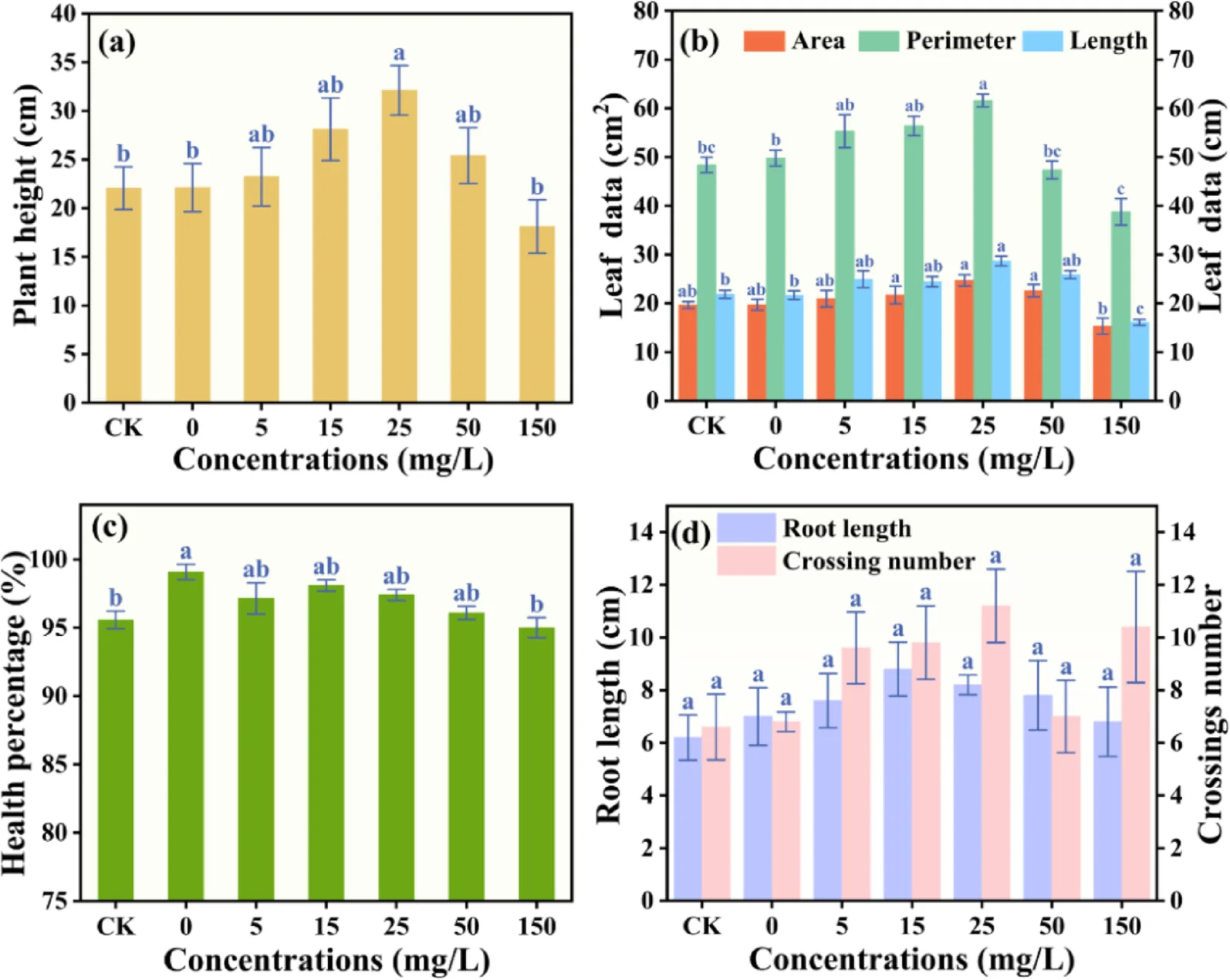
Effects of synthesized CQDs concentration on maize growth: **a** plant height, **b** leaf area, leaf perimeter, and leaf length, **c** percentage of healthy leaves, **d** root length, and root fork number. Error bars correspond to SD (n = 15) (Fig. **a**). Error bars correspond to SD (n = 5) (Fig. **b**–**d**). Marked with different letters indicating a significant difference (*p* < 0.05)

To investigate the effect of CQDs concentration on maize physiological parameters, aqueous CQDs solutions of varying concentrations were prepared and sprayed onto the leaves of maize plants at the seedling stage for ten consecutive days. The results were presented in Fig. 6. Figure 6a shows the average plant height of the experimental groups increased by 0.03%, 5.3%, 27.5%, 43.2%, 15.2%, and − 17.8% compared with the CK, respectively. Figure 6b shows that the average leaf length of the six experimental groups increased by − 0.8%, 12.3%, 14.1%, 31.2%, 18.4% and − 26.4% compared with the CK, respectively. The average leaf area of the six experimental groups increased by − 0.2%, 6.5%, 10.4%, 25.6%, 14.8%, and − 22.1% compared with the CK, respectively. The average leaf circumference of the six experimental groups increased by 2.8%, 14.3%, 16.5%, 27.3%, − 2.2%, and − 19.9% compared with the CK, respectively. Figure 6c indicates that foliar spraying of CQDs had a limited effect on maize plant health percentage. Figure 6d shows that the average root length of the experimental plants slightly increased by 12.9%, 22.6%, 41.9%, 32.3%, 25.8%, and 9.2% compared with the CK, respectively. The average number of root crossing increased by 2.9%, 45.5%, 48.5%, 69.7%, 6.1% and 57.6% compared with the CK, respectively. Data analysis indicated no significant differences in root length and root crossing count among groups, with considerable data dispersion. Based on the above findings, the physiological indicators of maize were most significantly improved when treated with a 25 mg/L CQDs aqueous solution via foliar spraying. Conversely, a higher concentration (150 mg/L) inhibited maize growth.

**Fig. 7:**
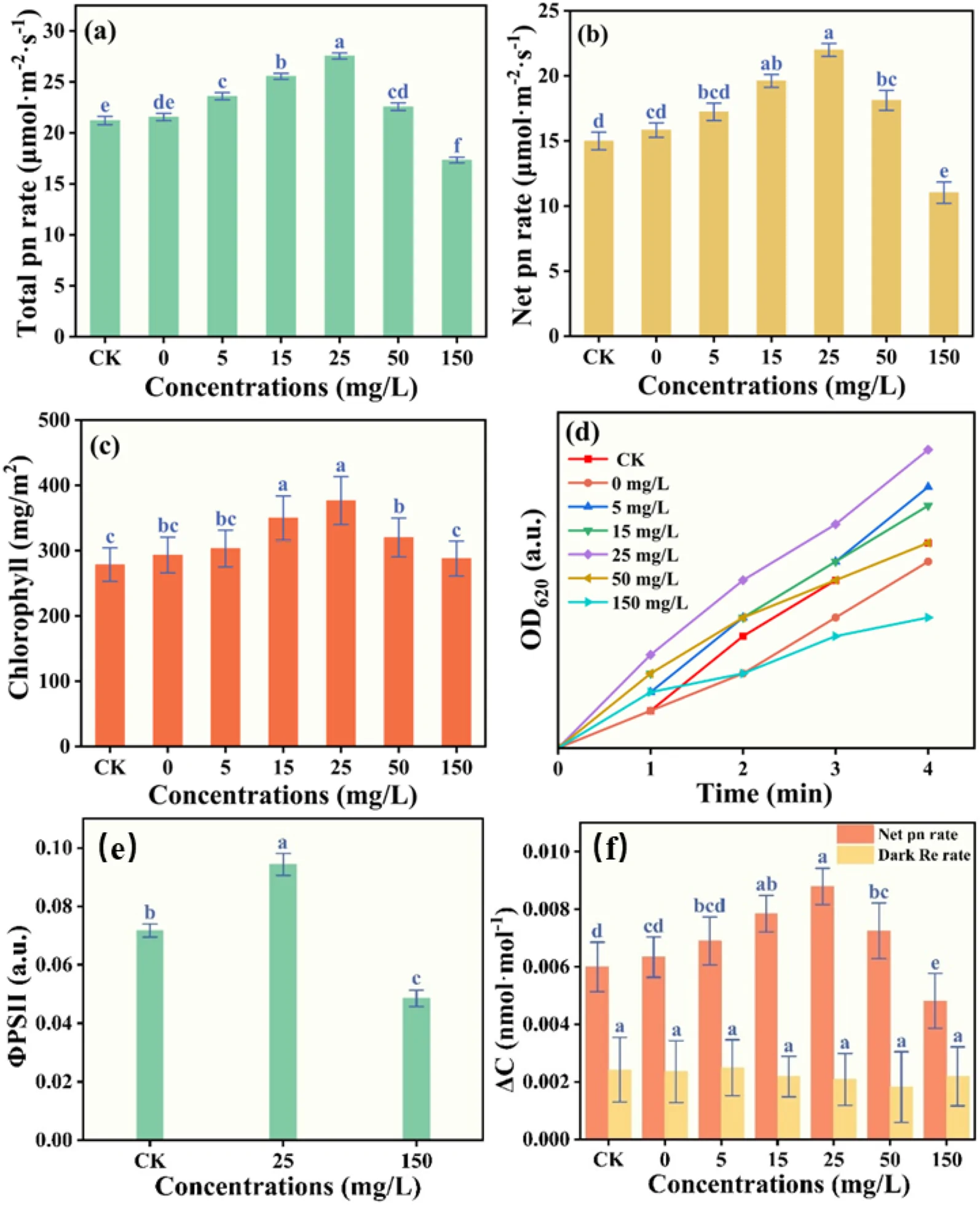
Effects of synthesized CQDs concentration on biochemical characteristics of maize: **a** total photosynthetic rate, **b** net photosynthetic rate, **c** chlorophyll content, **d** chloroplast Hill reaction activity. Error bars correspond to SD (n = 10). Marked with different letters indicating a significant difference (*p* < 0.05), **e** Photochemical efficiency at different CQD concentrations, **f** Changes in CO_2_ concentration under light and dark conditions at different CQD concentrations

Figure 7a shows the total photosynthetic rate of the six experimental groups increased by 1.6%, 11.3%, 20.5%, 29.8%, 6.2% and − 18.3% compared with the CK, respectively. Total photosynthetic rate is the sum of net photosynthetic rate and respiration rate. Figure 7b shows that the net photosynthetic rate of the six experimental groups increased by 5.6%, 14.9%, 30.8%, 46.6%, 20.8%, and − 26.5% compared with the CK, respectively. Figure 7c shows that the average chlorophyll content in the six groups increased by 5.3%, 8.9%, 25.7%, 35.2%, 14.9%, and 3.4%, respectively, compared with the CK. Chloroplast Hill reaction activity is also a crucial indicator. Figure 7d shows that after four minutes of light exposure, the Hill reaction activity of chloroplasts in the six experimental groups increased by − 9.1%, 27.3%, 18.2%, 45.5%, 0%, and − 36.4% compared to the CK, respectively. A clear concentration-dependent effect of CQDs on maize leaf photosynthesis was observed. The optimal concentration of 25 mg/L led to a significant increase in overall photosynthetic efficiency, chlorophyll levels, and chloroplast activity. Conversely, a higher concentration of 150 mg/L inhibited these photosynthetic functions. This trend was in full agreement with the prior physiological data obtained for maize. Figure 7e shows that ΦPSII increased at 25 mg/L CQDs but decreased at 150 mg/L, indicating a concentration-dependent promotion-inhibition transition. Figure 7f reveals that under illumination, CO_2_ depletion was greatest at 25 mg/L, whereas 150 mg/L suppressed CO_2_ assimilation; no differences were observed in the dark. These results suggest that moderate CQDs promote photosynthetic electron transport and CO_2_ fixation, while excessive concentrations inhibit them.

**Fig. 8:**
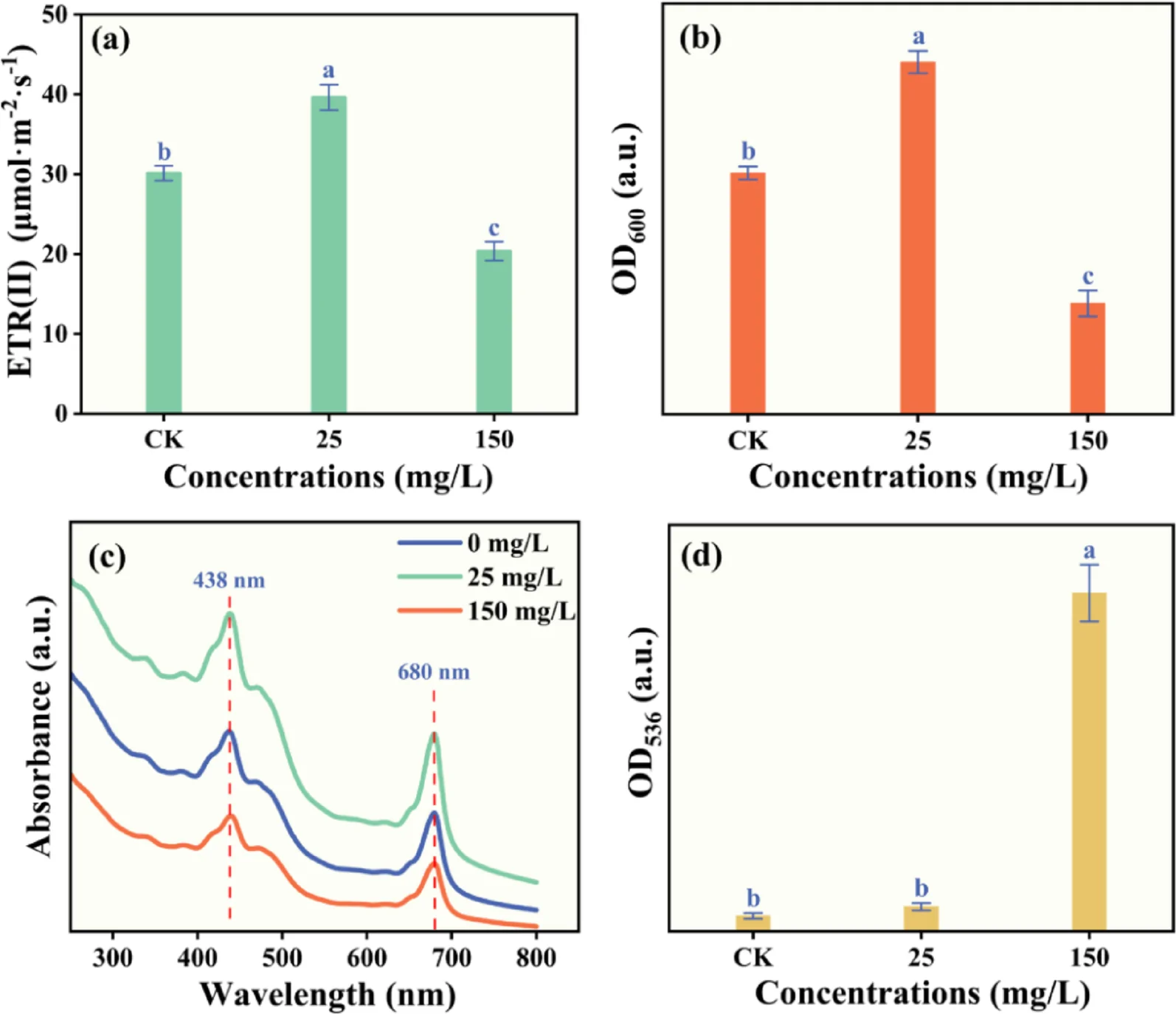
Mechanism of synthesized CQDs on maize photosynthesis: **a** chloroplast electron transport rate, **b** relative chloroplast electron transport rate, **c** chloroplast absorption spectrum, **d** hydroxyl radical content. Error bars correspond to SD (n = 3). Marked with different letters indicating a significant difference (*p* < 0.05)

To discuss the mechanism of photosynthetic enhancement by CQDs, three representative concentrations were selected based on the dose–response results established in Figs. 6 and 7: (i) 0 mg/L as the untreated control, (ii) 25 mg/L as the optimal concentration identified from growth/physiological assays, and (iii) 150 mg/L as the excessive dose exhibiting inhibitory effects, their effects on chloroplast light absorption and electron transport rate were investigated. The results were shown in Fig. 8. The chloroplast electron transport rate (ETR) and relative ETR in Fig. 8a and b were measured using the fluorescence module of a photosynthesis system and a commercial assay kit, respectively. The application of a 25 mg/L CQDs solution increased chloroplast ETR by 31.5%. It significantly elevated the relative ETR compared to CK, which could likely be due to the presence of numerous active sites facilitating electron transfer on the CQD surface. Figure 8c suggested that the pronounced increase in chloroplast light absorption induced by the 25 mg/L CQDs solution, relative to the control, was potentially due to the light-conversion mechanism of the CQDs. The underlying mechanism for the CQDs-induced promotion of maize growth and photosynthesis may be attributed primarily to the enhanced chloroplast electron transport rate and improved solar light absorption, aligning with previously published reports (Chowmasundaram et al. 2025; Liang et al. 2025). Conversely, it was also observed that a high concentration of CQDs aqueous solution induced notable reductions in both the chloroplast electron transport rate (ETR) and light absorption intensity. This inhibitory effect may be associated with the light-induced generation of a substantial amount of hydroxyl radicals (·OH) by high-concentration CQDs, as referenced in (Liu et al. 2021). Figure 8d revealed a marked increase in ·OH content under 150 mg/L CQDs treatment compared to CK, while the 25 mg/L treatment resulted in minimal change. Therefore, the photodegradation of high-concentration CQDs generates excessive ·OH, which in turn inhibits maize growth and photosynthesis.

**Fig. 9:**
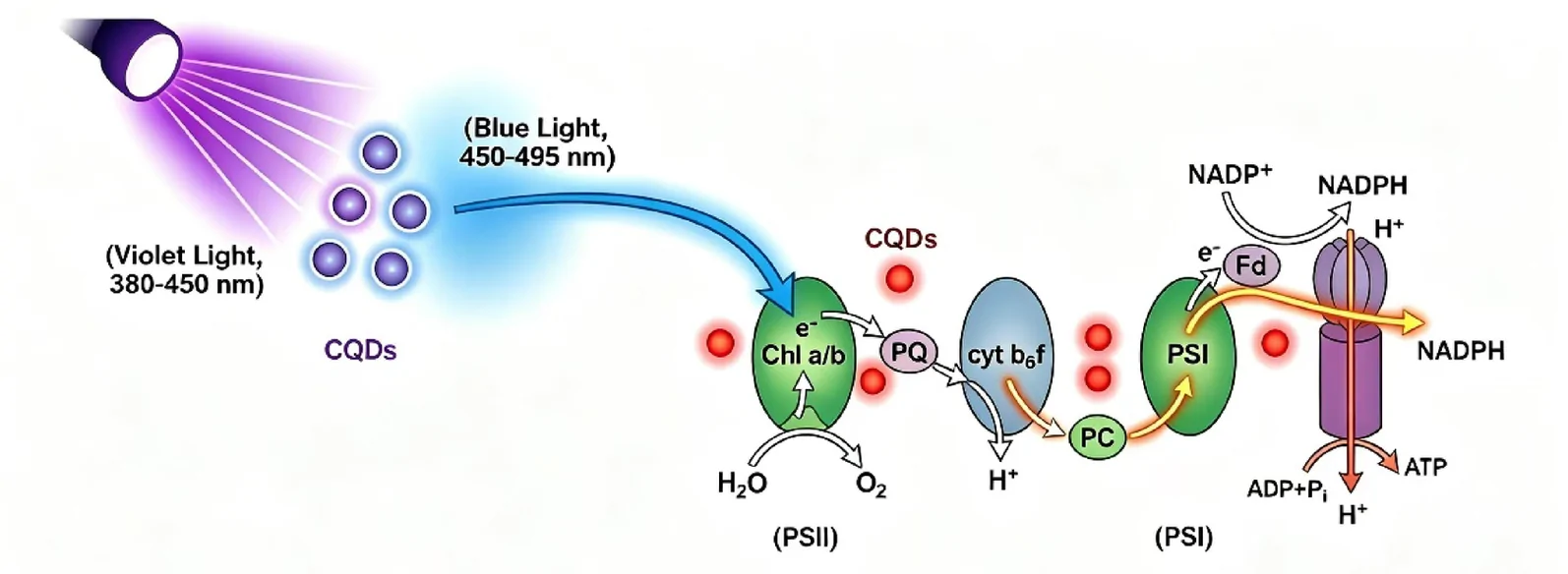
Possible mechanism diagram of photosynthesis enhancement

To further elucidate the underlying mechanism by which CQDs enhance photosynthesis in maize, a schematic diagram summarizing the proposed pathways is presented in Fig. 9. As illustrated, CQDs exert dual effects on the photosynthetic apparatus. On one hand, they function as spectral converters through a down-conversion process, absorbing incident ultraviolet/violet light and re-emitting it in the blue region (450–495 nm), which is more efficiently captured by photosynthetic pigments (Chl a/b). This spectral modulation effectively extends the photosynthetically active radiation (PAR) utilization range of the leaves. On the other hand, CQDs are proposed to directly participate in the thylakoid electron transport chain. Specifically, electrons derived from water oxidation at the oxygen-evolving complex of photosystem II (PSII) are sequentially transferred via plastoquinone (PQ), the cytochrome b_6_f complex (cyt b_6_f), and plastocyanin (PC) to photosystem I (PSI), ultimately driving the reduction of NADP⁺ to NADPH. Concurrently, the proton gradient established across the thylakoid membrane facilitates ATP synthesis via ATP synthase. The integration of these two synergistic pathways-spectral tuning and electron transport modulation-provides a plausible explanation for the observed increases in photochemical efficiency (ΦPSII), electron transport rate (ETR), and net CO₂ assimilation in CQD-treated maize leaves.

## Conclusions

This study employed agricultural waste maize stalks as a green precursor and utilized a mixed solvent of water and anhydrous ethanol to synthesize spherical water-soluble CQDs via hydrothermal synthesis. The diameter of CQDs was approximately 2.03 nm with a graphene structure, and the surfaces contained hydroxyl and carboxyl functional groups. The optimal excitation wavelength was determined to be 410 nm, with the maximum emission wavelength measured as 495 nm. Aqueous solutions of CQDs at various concentrations were sprayed onto the leaves of maize seedlings to investigate the concentration-dependent effects on plant growth. It was found that an optimal concentration of 25 mg/L significantly improved physiological parameters, including photosynthetic rate, chlorophyll content, and chloroplast activity. The mechanistic study revealed that CQDs promote photosynthesis through a dual pathway. On the one hand, coupling with chloroplasts enhanced the electron transport rate via the active sites on their surface. On the other hand, their light-conversion effect boosted light absorption by chloroplasts while concurrently protecting maize leaf cells from UV damage, thereby improving photosynthetic efficiency. Conversely, high concentrations of CQDs showed a detrimental effect on maize growth due to the excessive generation of ·OH via photodegradation. This study explores how carbon quantum dots (CQDs) affect maize photosynthesis, aiming to support their economical use in crop improvement and nano-agriculture. Unlike previous work, it synthesizes CQDs from corn stalks and applies them to maize to modulate photosynthesis.

## Data Availability

Data will be made available on request.
